# Severe early-onset osteoporosis due to heterozygous *WNT1* variants in adults: a clinical and therapeutic challenge

**DOI:** 10.1093/jbmr/zjaf150

**Published:** 2025-11-08

**Authors:** Eeva M Ryhänen, Riikka E Mäkitie, Tuula Pekkarinen, Heikki Kröger, Xiaoyu Tong, Liisa Kerttula, Outi Mäkitie, Camilla Schalin-Jäntti

**Affiliations:** Department of Endocrinology, Abdominal Center, Helsinki University Hospital and University of Helsinki, ENDO-ERN (European Reference Network on Rare Endocrine Conditions), 00290 Helsinki, Finland; Department of Otorhinolaryngology-Head and Neck Surgery, Helsinki University Hospital and University of Helsinki, 00290 Helsinki, Finland; Faculty of Medicine, University of Helsinki, 00290 Helsinki, Finland; Department of Endocrinology, Abdominal Center, Helsinki University Hospital and University of Helsinki, ENDO-ERN (European Reference Network on Rare Endocrine Conditions), 00290 Helsinki, Finland; Kuopio Musculoskeletal Research Unit (KMRU), Institute of Clinical Medicine, University of Eastern Finland, 70029 Kuopio, Finland; Department of Orthopedics, Kuopio University Hospital, 70029 Kuopio, Finland; Kuopio Musculoskeletal Research Unit (KMRU), Institute of Clinical Medicine, University of Eastern Finland, 70029 Kuopio, Finland; Diagnostic Center, Helsinki University Hospital and University of Helsinki, 00290 Helsinki, Finland; Children’s Hospital and Pediatric Research Center, University of Helsinki and Helsinki University Hospital, 00290 Helsinki, Finland; Research Program Unit, Research Program for Clinical and Molecular Metabolism, Faculty of Medicine, University of Helsinki, 00014 Helsinki, Finland; Genetics Research Program, Folkhälsan Research Center, 00014 Helsinki, Finland; Department of Molecular Medicine and Surgery, Karolinska Institutet and Clinical Genetics, Karolinska University Hospital, 171 77 Stockholm, Sweden; Department of Endocrinology, Abdominal Center, Helsinki University Hospital and University of Helsinki, ENDO-ERN (European Reference Network on Rare Endocrine Conditions), 00290 Helsinki, Finland

**Keywords:** Monogenic osteoporosis, vertebral fracture, WNTsignalling pathway, romosozumab, P4HB

## Abstract

Early-onset osteoporosis (EOOP) is diagnosed in premenopausal women or men under 50 yr of age when DXA-derived BMD is low (Z-score ≤ −2.0 or T-score ≤ −2.5) in the presence of a fragility fracture or a chronic disease. In young adult patients, it is essential to recognize EOOP and identify the underlying cause, including possible genetic defects, to optimize tailored treatments. Among monogenic causes, WNT1-related osteoporosis has been described in children and adults. We present two adults, a 27-yr-old male and his mother, who had no skeletal symptoms in childhood but presented as adults with back pain. Further studies revealed low BMD and multiple spinal fragility fractures, leading to rapid height loss and progressive kyphosis. Biochemistry was largely normal, but bone biopsies showed impaired bone metabolism with low bone turnover. Both were found to harbor a previously described pathogenic heterozygous variant in *WNT1*, which leads to impaired WNT signaling. We describe the challenges in their clinical care, sequence of treatments, and outcome. These patients highlight the value of a correct genetic diagnosis to better understand the mechanisms behind low bone mass and to enable early intervention. Furthermore, our study emphasizes the need for anabolic treatment options for both males and females with EOOP, and the importance of long-term treatment planning, including an exit strategy.

## Case description

*Patient 1, the proband*, a 27-yr-old Finnish male, was referred to a tertiary endocrinology center for back pain and thoracic vertebral fractures (Th7-9). He was previously healthy with normal growth and development in childhood and without long-term medications. A year earlier, he was diagnosed with hereditary spherocytosis as his hemoglobin was between 109-120 g/L (reference range 134-167). He had no history of smoking or heavy alcohol consumption.

**Figure 1:**
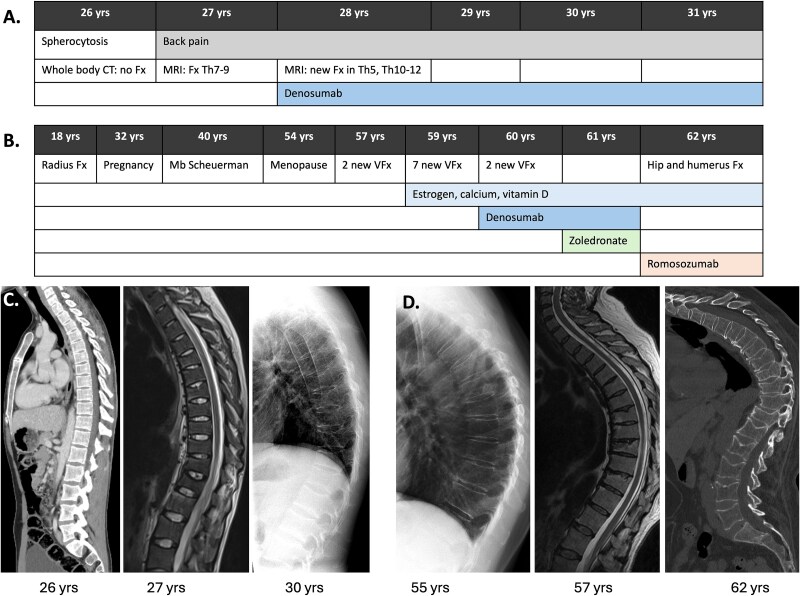
The timeline of symptoms, imaging findings and osteoporosis medications in patient 1 (A) and patient 2 (B). The sequence of radiological findings is presented for patient 1 (C) and for patient 2 (D). Fx, fracture; VFx, vertebral fracture.

He had no previously recorded fractures but prior to referral, had experienced intensive back pain. At initial presentation, MRI of thoracic and lumbar vertebrae showed possible fractures in Th7-9; these were not visible in the whole-body CT performed 2 yr earlier for the investigations of spherocytosis (Figure 1). DXA showed very low BMD in LS, FN, and TH (Z-scores −3.9, −2.5, and −2.8, respectively) (Table S1). No thoracic kyphosis was noted, and his sclerae and teeth were normal. There was no height loss; his height was 179 cm and weight 63 kg (BMI was 19.7 kg/m^2^).

During the following 6 mo, the proband sustained new vertebral fractures without trauma; radiographs of thoracic and lumbar vertebrae showed multiple vertebral fractures in four new vertebrae (Th5, Th10-12). He also lost 7.5 cm of his height in 6 mo. BMD further decreased 11.5% in L1-L4 and 1.6% in TH and 0.4% in FN from previous measurements (Table S1).

## Clinical problem

Patient’s skeletal disorder seemed to have a severely progressive course. Secondary causes for osteoporosis were not evident.

## Differential diagnosis and investigations

*In the proband*, secondary causes of osteoporosis were widely excluded. Biochemical data on calcium and phosphate metabolism were within reference ranges and markers of bone metabolism normal despite recent fractures (Table S1). In addition, 24-h urinary calcium was 5.82 mmol/24 h, serum tartrate resistant acid phosphatase 3.53 U/L, and FGF23 35 kRU/L (reference ranges: 1.3-6.5 mmol/24 h, 1.30-4.82 U/L, and 26-110 kRU/L, respectively). Hypercortisolism and hypogonadism were excluded. Whole-body CT, performed 2 yr earlier, fluorodeoxyglucose positron emission tomography (FDG-PET-CT) and bone marrow aspiration excluded inflammatory, hematologic or malignant diseases.

As no secondary causes of EOOP were present, genetic analyses were undertaken. A comprehensive panel covering 38 genes linked to skeletal fragility was performed. In addition to sequence changes, the analysis also detects deletions and duplications that involve two or more contiguous exons or span multiple genes overlapping the panel genes or phenotypically relevant genes. The following genes were analyzed (Fulgent Genetics): *ALPL*, *ANO5*, *B4GALT7*, *BMP1*, *CLCN5*, *COL1A1*, *COL1A2*, *CREB3L1*, *CRTAP*, *DMP1*, *ENPP1*, *FGF23*, *FKBP10*, *GNAS*, *GORAB*, *IFITM5*, *LMNA*, *LRP5*, *MAFB*, *MMP2*, *NOTCH2*, *P3H1*, *P4HB*, *PHEX*, *PLOD2*, *PLS3*, *PPIB*, *SEC24D*, *SERPINF1*, *SERPINH1*, *SLC34A3*, *SP7*, *SPARC*, *TAPT1*, *TENT5A*, *TMEM38B*, *TNFRSF11A*, and *WNT1*.

The genetic test revealed a heterozygous pathogenic variant in exon 4 of *WNT1* (p.Cys218Gly) in chromosome 12. The same variant has been reported in the heterozygous state in several individuals in two other unrelated Finnish families with early-onset osteoporosis (EOOP).^1^^,^^2^ The variant is predicted to lead to the substitution of cysteine to glycine, resulting in impaired capacity to induce canonical WNT signaling, its target genes and subsequently bone mineralization. It is reported in the Single Nucleotide Polymorphism database (dbSNP) and ClinVar and classified as a disease-causing mutation in the Human Gene Mutation Database (HGDB).

In addition, the proband also harbored a heterozygous variant in exon 7 of *P4HB* (p.Lys308Arg). The variant is absent in the general population but not listed in HGDB. It is predicted to result in a single amino acid substitution of lysine to arginine. As the physiochemical difference between these two amino acids measured by Grantham’s Distance was 26, it was considered a conservative change, that is, the two amino acids are similar in physiochemical properties. Thus, the variant was classified as a variant of unknown significance (VUS).

**Figure 2:**
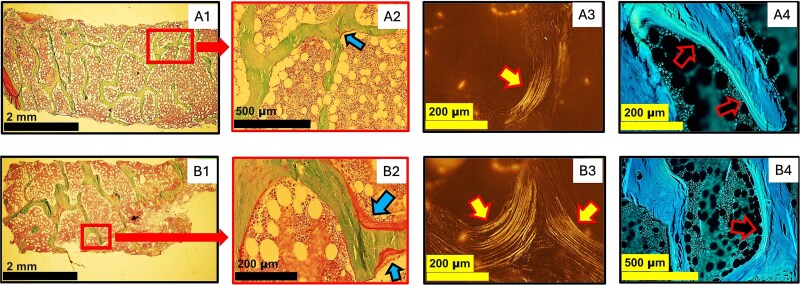
Bone histomorphometric analyses of patient 1 (upper panel) and patient 2 (lower panel). Typical microarchitectures of cancellous bone of iliac crest specimens under light microscopy (A1 and 2; B1 and 2), polarization microscopy (A3 and B3), and fluorescence/UV microscopy (A4 and B4) are exemplified by our case 1 (male, age 29 yr) and case 2 (female, age 62 yr). The magnified images (highlighted by rectangles in A1 and B1) demonstrate the unmineralized bone tissue (osteoid, indicated by arrows in A2 and B2). The parallel-organized osseous lamellae are indicated by arrows in A3 and B3 in polarization view and tetracycline double labels are indicated by arrows in A4 and B4 in UV view. Masson–Goldner trichrome stain (A1 and 2; B1 and 2), magnification 20x (A1 and B1); 100x (A2 and B4); 200x (A3 and 4; B2 and 3).

As the proband’s skeletal phenotype was more severe than those reported earlier for WNT1-related osteoporosis, bone biopsy was obtained to further evaluate bone quality. Vertical biopsy was taken under local anesthesia from the anterior iliac crest. To assess bone turnover, fluorochrome double labeling was performed with tetracycline and a 3:10:3:5-d schedule. Quantitative bone histomorphometry showed low bone turnover, low trabecular bone volume, and thin trabeculae. The amount of osteoid was low, active osteoblasts absent, and bone resorption decreased indicating low bone turnover osteoporosis. The presence of double labels in UV microscopy excluded mineralization defect (Figure 2, Table S3).

*Patient 2*, *mother of the proband*, is a 59-yr-old Caucasian woman with a history of years-long continuous, worsening, and incapacitating back pain. She had no previously diagnosed bone-affecting long-term illnesses or regular medications, no history of smoking or alcohol use. She had had a distal radius fracture at the age of 18 and was diagnosed with Mb Scheuermann-like vertebrae at age 40. She experienced menopause at 54 yr of age. She had experienced several episodes of severe back pain after lifting weights or carrying backpacks. Spinal radiograph taken after falling off a bicycle 5 yr prior to the diagnosis showed only thoracic kyphosis but no fractures.

An MRI, taken 3 yr prior to the diagnosis, showed for the first time old compression fractures in Th10 and Th12 (Figure 1). A second MRI 2 yr later showed additional non-traumatic fractures in altogether 9 vertebrae (Th9-13 and L2-5) with up to 50% height reduction in Th11 and Th12. DXA, measured then for the first time, showed osteoporosis in LS, FN, and TH (Table S2). She commenced treatment with 1000 mg of calcium carbonate and 70 μg of vitamin D3 daily, and transdermal ethinyl estradiol 50 μg twice a week to improve BMD. She also started physiotherapy and duloxetin 30-60 mg daily to ease her back pain.

Thereafter, patient 2 was also referred to the same tertiary endocrinology unit as her son for her osteoporosis and multiple vertebral fractures. Her height at the time was 157.2 cm (maximal height 171.5 cm, −14.3 cm) and weight 70 kg (BMI 28.3 mg/m^2^). Degree of thoracic kyphosis in a spinal radiograph was 74 grades and MRI showed new compression fractures in vertebrae Th 6-8 and L1; fractures were now present in all vertebrae from Th6 to L5 (Figure 1). Trabecular bone score, which assesses the amount and quality of trabecular bone, was reduced (Table S2). Biochemistry regarding secondary causes of osteoporosis remained normal. Her father had sustained a hip fracture at the age of 76 yr and the paternal grandmother had had severe kyphosis, both since deceased. Her son was the only in her family with osteoporotic fracture in young age.

Genetic panel covering 38 genes with deletion and duplication analysis revealed one heterozygous pathogenic mutation: the same *WNT1* variant as in her son (p.Cys218Gly) but not the *P4HB* variant.

Quantitative bone histomorphometry of iliac crest biopsy showed low trabecular bone volume and reduced bone resorption. The amount of osteoid was within normal range and UV-microscopy showed double labels with no signs of impaired mineralization (Figure 2, Table S3).

## Diagnosis

WNT1-related EOOP due to a heterozygous missense variant p.Cys218Gly in exon 4 of *WNT1*.

## Treatment and progression

*The proband* was initiated with denosumab, a monoclonal antibody for RANKL, with 60 mg every 6 mo as soon as the results of genetic testing and bone biopsy were available (Figure 1). Teriparatide was considered as an adjunct, but prior studies regarding teriparatide in WNT1 patients^3^ were not in support nor did the patient consent. Bone turnover markers decreased during the use of denosumab (S-P1NP decreased from 38 to 11 μg/L and fP-CTx from 0.49 to 0.03 μg/L) (reference ranges: 15-59 and 0.24-1.2 μg/L, respectively) (Table S1). Persistent back pain and the patient’s fear of recognizing new fractures have prevented further DXA-measurements. Therefore, since the initiation of denosumab, BMD has been measured only from 33% radius (absolute BMD 0.761 cm/m^2^, Z-score 1.0 and T-score 1.1). No new vertebral fractures have been documented in spinal radiographs during the 34-mo follow-up, and he continues with denosumab. Due to severe back pain, his walking is limited to only short distances.

*Patient 2*, *mother of the proband*, also initiated denosumab 60 mg subcutaneously every 6 mo at the time of diagnosis. After 15 mo, she fell and sustained a pertrochanteric hip fracture, as well as fractures to proximal humerus and fifth metacarpal. The hip fracture was operated on, other fractures were treated conservatively. Eighteen months later, DXA showed 13% improvement in LS, 5.1% in FN, and 3.9% in TH compared with the first DXA 2 yr earlier (Table S2).

Despite this initial improvement, BMD remained low, she fractured during denosumab therapy and had severe spinal osteoporosis. Therefore, denosumab treatment was discontinued. Intravenous zoledronate 5 mg was given 6 mo after the last denosumab injection to prevent rebound after discontinuation of denosumab. An osteoanabolic treatment, romosozumab was initiated 9 mo after the zoledronate infusion with 210 mg subcutaneously monthly (Figure 1). S-P1NP rose as expected after one month of treatment (from 34 to 84 μg/L, reference range: 15-59) and returned to baseline level. At 6 mo, DXA showed 16.4% increase in FN T-score, 0.6% decrease in LS, and 1.2% increase in TH, compared with DXA performed 9 mo earlier (Table S2).

Father of proband, a 74-yr-old Caucasian male, was shown in segregation analysis to harbor the same *P4HB* variant but not the *WNT1* variant. His BMD was evaluated to assess the possible impact of the *P4HB* variant on the bone phenotype. He had no history of fractures, bone deformities, or other clinical manifestations typically seen in Cole–Carpenter syndrome,^4^ which is caused by heterozygous *P4HB* variants. DXA showed osteopenia: T-scores in FN, TH, and LS were −2.1, −1.5, and −1.9, respectively. Apart from a mild vitamin D deficiency (S-25OHD 42 nmol/L, reference range over 50 nmol/L), concentrations of serum calcium, phosphate, and bone turnover markers (P1NP and fP-CTx) were in the reference range.

## Discussion

Early-onset osteoporosis (EOOP) is characterized by both low BMD and either a bone fracture or a predisposing chronic disease.^5^ When secondary causes of EOOP are not present, genetic causes are advised to be evaluated. With vigorous research and growing knowledge on the genetics underlying idiopathic osteoporosis, familial forms of osteoporosis are nowadays diagnosed not only in pediatric patients but also in adults.^5^

Our finding of severe EOOP in result of a heterozygous *WNT1* variant is new. Earlier studies have established that biallelic *WNT1* variants cause Osteogenesis Imperfecta Type XV characterized by multiple fractures at a very young age, bone deformities, and extraskeletal manifestations of OI.^1^^,^^2^ Patients with heterozygous *WNT1* variants, on the other hand, portray a milder phenotype with low BMD and EOOP without extraskeletal characteristics. Despite some long-bone fractures in childhood, the vertebral fractures usually manifest after age 50 yr.^6^ In contrast, our 27-yr-old proband and his 59-yr-old mother had severe osteoporosis with multiple fragility fractures and very low BMD despite harboring the same *WNT1* variant in a heterozygous state. Both suffered from chronic, debilitating back pain. The mother had multiple, progressive fragility fractures in her thoracic spine leading to severe kyphosis and a 14-cm loss in adult height. The son lost 7.5 cm of his adult height only in 6 mo.

Based on our current understanding, and as underlined by the two patients presented here, the possibility of monogenic osteoporosis should be considered in patients with severe primary osteoporosis of unknown cause. In a recent study on two cohorts of patients with idiopathic osteoporosis, an underlying pathogenic variant was detected in 42% of children and 19% of adults.^6^^,^^7^ Though still rare, *WNT1* variants are found in up to 6%-10% of children, premenopausal women, and <50-yr-old males diagnosed with idiopathic osteoporosis^6^ and reported to explain 8%-10% of idiopathic EOOP in premenopausal women, men aged <50 yr, or individuals with fragility fractures at an early age.^7^ Our patients highlight the need to assess for hereditary forms of osteoporosis also in adult patients with idiopathic osteoporosis.

The clinical phenotype in hereditary osteoporosis is highly variable, even among family members or close relatives despite carrying the same identical mutation. Our male index had a more severe phenotype than his mother with an early onset and aggressive and progressive disease course. Several lifestyle and environmental factors, such as nutritional factors, physical activity, and hormonal status, contribute to an individual’s bone health and may predispose to compromised bone strength. Furthermore, despite women being commonly more prone to impaired BMD, prior studies have reported observations of an offspring harboring a more aggressive phenotype than their mutation-positive mother. In light of this, despite their shared genetic background, it is possible that additional factors, including potentially even impaired bone mass gain during fetal development, contribute to the differences seen between our two patients.

Despite harboring a disease-causing genetic variant since birth, monogenic forms of EOOP may have rapid onset and progression only later. In our index patient, skeletal growth was markedly impaired possibly due to the *WNT1* mutation and low calcium diet, but only led to fractures in early adulthood. It remains uncertain whether the sudden onset of fractures is a consequence of subnormal peak bone mass accrual, impaired maintenance of bone mass, or represents a fracture cascade triggered by the first trauma-related vertebral fracture.^8^ In previous cases of WNT1 osteoporosis, the first fractures have been lactation-induced.^1^^,^^9^ In the mother, fractures may have ensued more slowly over a longer period of time, as her first spinal radiograph may not have been sensitive enough to disclose true compression fractures.

We acknowledge that the secondary *P4HB* variant observed in our index patient may have contributed to his severe skeletal phenotype. Heterozygous pathogenic *P4HB* variants are associated with Cole–Carpenter syndrome (CLCRP1), which is an OI-like disorder diagnosed in early childhood with multiple fractures and extraskeletal manifestations, such as craniosynostosis, bone deformities, and ocular proptosis.^4^ The gene encodes protein disulfide isomerase (PDI), involved in post-translational modification of collagen I. Alterations in the conformational form of PDI and subsequent accumulation of dysfunctional PDI protein causes stress in endoplasmic reticulum with potential cell toxicity to osteoblasts.^4^ In our patient, the genomic change in *P4HB* was classified as a VUS and considered a conservative change not altering the physiochemical properties of PDI. Furthermore, his father, carrier of the same variant, did not have signs of CCS and his BMD was not particularly impaired. We therefore consider this variant to be of low significance to the index’s skeletal pathology, but its true nature remains undetermined.

The proband was also diagnosed with hereditary spherocytosis. A possible additional negative effect of this disease cannot be excluded as a small case-control study showed reduced BMD and vitamin D concentration in children with spherocytosis.^10^

Despite great research efforts, the management of WNT1-related osteoporosis remains challenging. In general, current guidelines for postmenopausal osteoporosis management encourage toward a long-term plan when using osteoporotic medications, especially in severe osteoporosis.^10^ Previous use of antiresorptive agents can inhibit the effect of anabolic bone medications later.^10^ Bisphosphonates are not optimal in low-turnover osteoporosis. While the first osteoanabolic agent teriparatide in postmenopausal women increases BMD in trabecular bone, it can decrease cortical bone density. In prior WNT1 patients, teriparatide treatment has been described to increase BMD and bone turnover markers, but to lesser extent than in postmenopausal osteoporosis. However, our previous study evaluating teriparatide’s effects on the bone tissue level by bone histomorphometry^3^ suggests that teriparatide has very little effect on bone mineral content or dynamic indices of bone formation although the size of osteocyte lacunae decreases. Despite proven benefits of denosumab in postmenopausal osteoporosis, its impact in WNT1 osteoporosis remains unclear.

Given its central role in bone metabolism by activating osteoblasts and promoting osteoclastogenesis, the canonical WNT1 pathway has become a key target for novel osteoporosis therapeutics. The pathway’s main tissue-specific negative regulator is sclerostin, which is secreted by mature osteocytes surrounded in mineralized matrix. Sclerostin inhibits osteoblast function and bone formation and promotes osteoclast activity.^12^ Romosozumab is a fully humanized immunoglobulin G2 monoclonal antibody neutralizing the action of sclerostin,^9^ thus activating canonical WNT pathway, increasing osteoblast activity and reducing osteoclast activity through osteoclast regulators. It was approved by the Food and Drug Administration in 2019 and by European Medicines Agency in 2020 as an osteoanabolic agent for the management of osteoporosis in humans. Research on romosozumab in WNT1 osteoporosis; however, remains scarce. Romosozumab has shown to significantly improve BMD in two previously described patients with a different *WNT1* mutation and subsequently very low BMD and multiple fractures: a 40-yr-old treatment-naïve man and a 28-yr-old woman with prior teriparatide and denosumab managements.^8^^,^^13^

An osteoanabolic medication is the first-choice treatment in low bone turnover. However, when the osteoporosis medication was initiated for our patients, romosozumab was not yet available in Finland. In the mother, major osteoporotic fractures continued during treatment with denosumab. Hence, treatment was changed to WNT-targeted osteoanabolic agent romosozumab, and at already 6 mo improvement was seen in FN (+16.4% compared with previous DXA), but not in LS (−0.6%). In contrast to the female patient reported earlier,^8^ it is possible that the antiresorptive medication our patient used prior to commencement with romosozumab may have reduced the potential increase in BMD in trabecular bone.

The patients’ osteoporosis medication needs to be terminated within the next few years, and an exit strategy needs to be planned. For the son, to further improve BMD an osteoanabolic medication (ie, teriparatide) could be combined with denosumab for 2 yr. Romosozumab is not indicated for men in Europe. After that, zoledronic acid should be given twice with 3-6 mo interval as current guideline suggests for denosumab cessation.^14^

The mother will also need bisphosphonate directly after the 12 mo course of romosozumab or she can continue with denosumab. In that case, she would also need two zoledronic acid infusion with 3-6 mo interval. However, this strategy may not prevent vertebral fractures in these high-risk patients. Therefore, in the future, we may consider overlapping, combined treatment approaches with denosumab and romosozumab as reported recently.^15^^,^^16^ In future, larger prospective controlled studies are needed to find an effective exit strategy for denosumab.

## Unanswered questions

The determinants of clinical severity, which varies between individuals with identical mutations, are unclear. With better understanding of the pathophysiology underlying different forms of hereditary osteoporosis, the role and optimal choice of each osteoporosis medication will become more accurate. Romosozumab may serve as a relevant first-line option when initiating medication in low-turnover osteoporosis. While bone biopsy remains an informative tool in analyzing bone remodeling, additional non-invasive modalities are needed to assess bone turnover and histology in complex and severe bone diseases.

## Conclusions and future directions

We present two related patients with low BMD and rapidly progressive spinal osteoporosis with multiple vertebral fractures due to a heterozygous *WNT1* mutation. Our patients’ disease onset at an unusually early age (the son) and the severe disease progression prompted genetic investigations. An exact genetic diagnosis of EOOP enables better understanding of the disease mechanisms and the causes of low bone mass. Consequently, this enables early intervention, selection of an optimal treatment, and long-term management planning. The improved access to an osteoanabolic treatment directly targeting the WNT signaling pathway is an encouraging development, but treatment access is still limited. However, evidence-based treatment guidelines are lacking for these rare monogenic forms of osteoporosis and treatment results need to be evaluated in future studies.

## Supplementary Material

Ryhanen_Supplemental_Material_zjaf150

## Data Availability

The data underlying this article are available in the article and in its online supplementary material.
