# Supplementary material for: Severe early-onset osteoporosis due to heterozygous WNT1 variants in adults: a clinical and therapeutic challenge
Source: J Bone Miner Res. 2025 Nov 8;41(1):85–90. doi: 10.1093/jbmr/zjaf150 (PMC12765685; doi:10.1093/jbmr/zjaf150)
Supplement: Ryhanen_Supplemental_Material_zjaf150 [file ryhanen_supplemental_material_zjaf150.docx]

**Severe early-onset osteoporosis due to heterozygous *WNT1* variants in adults: A clinical and therapeutic challenge**

Ryhänen EM^1^, Mäkitie RE^2,3^, Pekkarinen T^1^, Kröger H^4,5^, Tong X^4^, Kerttula L^6^, Mäkitie O^7,8,9,10*^, Schalin-Jäntti C^1*^

^1^Endocrinology, Abdominal Center, Helsinki University Hospital and University of Helsinki, ENDO-ERN (European Reference Network on Rare Endocrine Conditions), Helsinki, Finland

^2^Department of Otorhinolaryngology - Head and Neck Surgery, Helsinki University Hospital and University of Helsinki, Helsinki, Finland

^3^Faculty of Medicine, University of Helsinki, Helsinki, Finland

^4^Kuopio Musculoskeletal Research Unit (KMRU), Institute of Clinical Medicine, University of Eastern Finland Kuopio, Finland

^5^Department of Orthopedics, Kuopio University Hospital, Kuopio, Finland

^6^Diagnostic Center, Helsinki University Hospital and University of Helsinki.

^7^Children’s Hospital, Pediatric Research Center, University of Helsinki and Helsinki University Hospital, Helsinki, Finland

^8^Research Program for Clinical and Molecular Metabolism, Faculty of Medicine, University of Helsinki, Helsinki, Finland

^9^Folkhälsan Research Center, Helsinki, Finland

^10^Department of Molecular Medicine and Surgery, Karolinska Institutet and Clinical Genetics, Karolinska University Hospital, Stockholm, Sweden

*shared last authorship

**Corresponding author:**

Outi Mäkitie, MD PhD

Folkhälsan Research Center

P.O. Box 63

FI-00014 University of Helsinki

Finland

[outi.makitie@helsinki.fi](mailto:outi.makitie@helsinki.fi)

tel. +358-442050155

**Supplemental Table 1.** Biochemical values and results of DXA assessments at baseline, after 6 months of follow up and after 6 months of Denosumab treatment in Patient 1.

|  | **Patient 1** | | | **Reference range** |
| --- | --- | --- | --- | --- |
|  | **Baseline** | **+6 months** | **+6 months ofDMAB** |  |
| Ca-ion (mmol/L) | 1.23 | 1.18 |  | 1.15-1.30 |
| PTH (ng/L) | 48 | 47 |  | 18–80 |
| Pi (mmol/L) | 0.95 | 0.99 |  | 0.76–1.41 |
| Crea (µmol/L) | 62 | 65 |  | 50-90 |
| ALP (U/L) | 62 | 82 |  | 35–105 |
| 25-OHD (nmol/L) | 59 | 74 |  | >50 |
| P1NP (µg/L) | 38 | 21 | 11 | 15-59 |
| CTx (µg/L) | - | 0.49 | 0.03 | 0.24-1.2 |
| **Lumbar spine L1-L4** |  |  | NA |  |
| BMD (g/cm^2^) | 0.700 | 0.618 |  |  |
| Z-score | -3.9 | -4.6 |  |  |
| **Total hip** |  |  | NA |  |
| BMD (g/cm^2^) | 0.699 | 0.690 |  |  |
| Z-score | -2.8 | -2.9 |  |  |
| **Femoral neck** |  |  | NA |  |
| BMD (g/cm^2^) | 0.718 | 0.715 |  |  |
| Z-score | -2.5 | -2.6 |  |  |

**Supplemental Table 2.** Applied osteoporosis treatment, bone turnover markers and results of DXA assessments at baseline and during various osteoporosis therapies in Patient 2.

|  | **Baseline** | **6 months** | **24 months** | **33 months** | **39 months** |
| --- | --- | --- | --- | --- | --- |
| **Medication** | Estrogen initiated | DMAB initiated | 18 months of DMAB;  DMAB discontinued, ZOL initiated | 9 months of ZOL; ZOL discontinued, ROMO initiated | 6 months of ROMO |
| P1NP (µg/L) | - | 40 | 52 | 38 | 43 |
| CTx (µg/L) | - | 0.37 | 0.14 | 0.26 | 0.20 |
| **Lumbar spine L3-L4** |  |  |  |  |  |
| BMD (g/cm^2^) | 0.752 | 0. 797 | 0.851 | 0.897 | 0.902 |
| T-score | -3.2 | -3.2 | -2.3 | -1.9 | -1.8 |
| **Total hip** |  |  |  |  |  |
| BMD (g/cm^2^) | 0.686 | 0.702 | 0.708 | 0.711 | 0.719 |
| T-score | -2.1 | -2.0 | -1.9 | -1.9 | -1.8 |
| **Femoral neck** |  |  |  |  |  |
| BMD (g/cm^2^) | 0.552 | 0.573 | 0.581 | 0.556 | 0.648 |
| T-score | -2.7 | -2.5 | -2.4 | -2.6 | -1.8 |
| **Trabecular Bone Score** | - | 1.118 | 1.272 | 1.213 | - |

**Supplemental Table 3**. Bone histomorphometric findings in Patient 1 and Patient 2 at baseline. Values outside the reference range are in bold

| **Parameter** | **Patient 1**  **(27 yrs old male)** | **Reference range^a^** | **Patient 2**  **(59 yrs old female)** | **Reference range^b^** |
| --- | --- | --- | --- | --- |
| BV/TV (%) | **13.3** | 18.9 - 28.9 | 16.3 | 15.9 - 26.3 |
| OV/BV (%) | 0.99 | 1.14 - 2.4 | **2.7** | 0.72 - 2.1 |
| OS/BS (%) | **6.5** | 10.8 - 21.4 | 13.4 | 5.67 - 16.97 |
| ES/BS (%) | 2.9 | 2.5 - 4.9 | **0.46** | 1.71 - 6.11 |
| ObS/BS (%) | **0.0** | 3.4 - 7.4 | 4.3 | 3.69 - 11.91 |
| OcS/BS (%) | 0.35 | 0.3 - 0.6 | **0.0** | 0.05 - 0.83 |
| MS/BS (%) | **1.8** | 5.9 - 9.7 | 4.9 | 2.15 - 8.47 |
| MAR (µm/d) | 0.69 | 0.52 – 0.76 | 0.67 | - 1. – 0.67 |

BV = bone volume, TV = tissue volume, OV = osteoid volume, OS = osteoid surface, BS = bone surface, ObS = osteoblast surface, OcS = osteoclast surface, O.Th = osteoid thickness, MS = mineralizing surface.

^a^Rehman MT, Hoyland JA, Denton J, Freemont AJ. Age related histomorphometric changes in bone in normal British men and women. J Clin Pathol 1994: 47(6):529-34

^b^Recker RR, Lappe JM, Davies M, Kimmel D. Perimenopausal bone histomorphometry before and after menopause. Bone 2018:108:55-61
